# Congenital partial pericardial defect discovered incidentally during surgery for lung cancer: a case report and literature review

**DOI:** 10.1186/s12893-021-01453-3

**Published:** 2021-12-31

**Authors:** Yoshihito Iijima, Masahito Ishikawa, Shun Iwai, Aika Yamagata, Nozomu Motono, Shigeki Yamagishi, Kiyoshi Koizumi, Hidetaka Uramoto

**Affiliations:** 1 Department of Thoracic Surgery, Kanazawa Medical University, 1-1 Daigaku, Uchinada-machi, Kahoku-gun, Ishikawa 920-0293 Japan; 2 Department of Thoracic Surgery, Aidu Chuo Hospital, Fukushima, Japan

**Keywords:** Congenital pericardial defect, Primary lung cancer, Video assisted thoracic surgery

## Abstract

**Background:**

Congenital pericardial defects are rare congenital anomalies, often asymptomatic and incidentally detected during thoracic surgery.

**Case presentation:**

A 74-year-old man with primary lung cancer (cT1cN0M0, Stage IA3) underwent thoracoscopic radical lobectomy. At the time of thoracotomy, a pericardial defect was found on the ventral side of the hilar region, and the left atrial appendage was exposed. Due to concern that contact between the bronchial stump and the left atrial appendage may lead to postoperative bleeding and heart hernia, the pericardial defect was closed with an expanded polytetrafluoroethylene GoreTex® membrane. Preoperative computed tomography was reanalyzed with a 1 mm slice, congenital pericardial defect was detected as the pericardium had penetrated under the left atrial appendage.

**Conclusions:**

In congenital partial pericardial defect, contact between the left atrial appendage and bronchial stump, due to movement of the lung or heart, increases the risk of bleeding after lung resection. Therefore, closure of the defect should be considered. Although it is difficult to diagnose congenital partial pericardial defect preoperatively, computed tomography taken with a slice thickness of 1 mm is useful for diagnosis.

## Background

Congenital pericardial defect (CPD) is a rare anomaly that refers to congenital absence of the pericardium. It can occur as a complete absence of the entire pericardium, the right or left portion of the pericardium or a partial, foramen-like defect of the right or left pericardium [1]. The frequency of its occurrence is reported to be 0.01–0.04% [1, 2]. Repair of congenital partial pericardial defects (CPPD) is controversial. Generally, if there are no symptoms due to a pericardial defect, surgical treatment is not required [3]. On the other hand, lobectomy or pneumonectomy reduce the support to the mediastinal organs and can cause cardiac arrest or myocardial ischemia due to cardiac hernia [4, 5]. Therefore, closure of the defect should be considered. Diagnosing CPPD preoperatively is associated with reduced risk of cardiac injury [3]. In pulmonary surgery, complications of pericardial emphysema secondary to pneumothorax are often associated with preoperative diagnosis of CPPD. We report a case of a CPPD that was incidentally diagnosed during surgery for upper left lobe lung cancer, and describe the use fulness of 1 mm sliced CT for the diagnosis of CPPD.

## Case presentation

Written, informed consent was obtained from the patient for the publication of this report and its accompanying images.

**Fig. 1:**
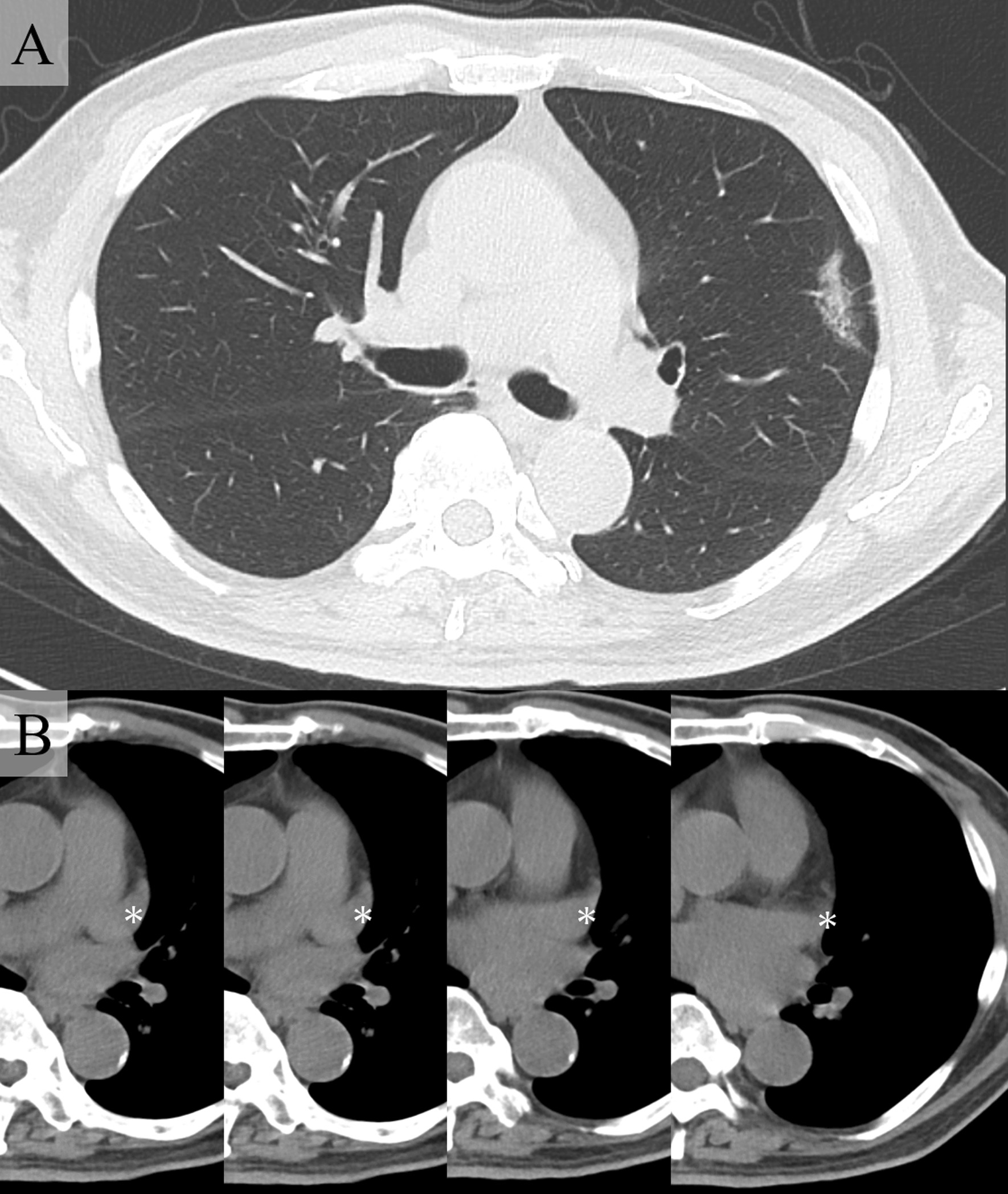
Computed tomography imaging. **A** A ground glass shadow with a maximum tumor diameter of 32 mm and a solid component diameter of 24 mm in the left lung S3. **B** No abnormal findings seen in the cardiac shadow (white asterisk indicates left atrial appendage)

**Fig. 2:**
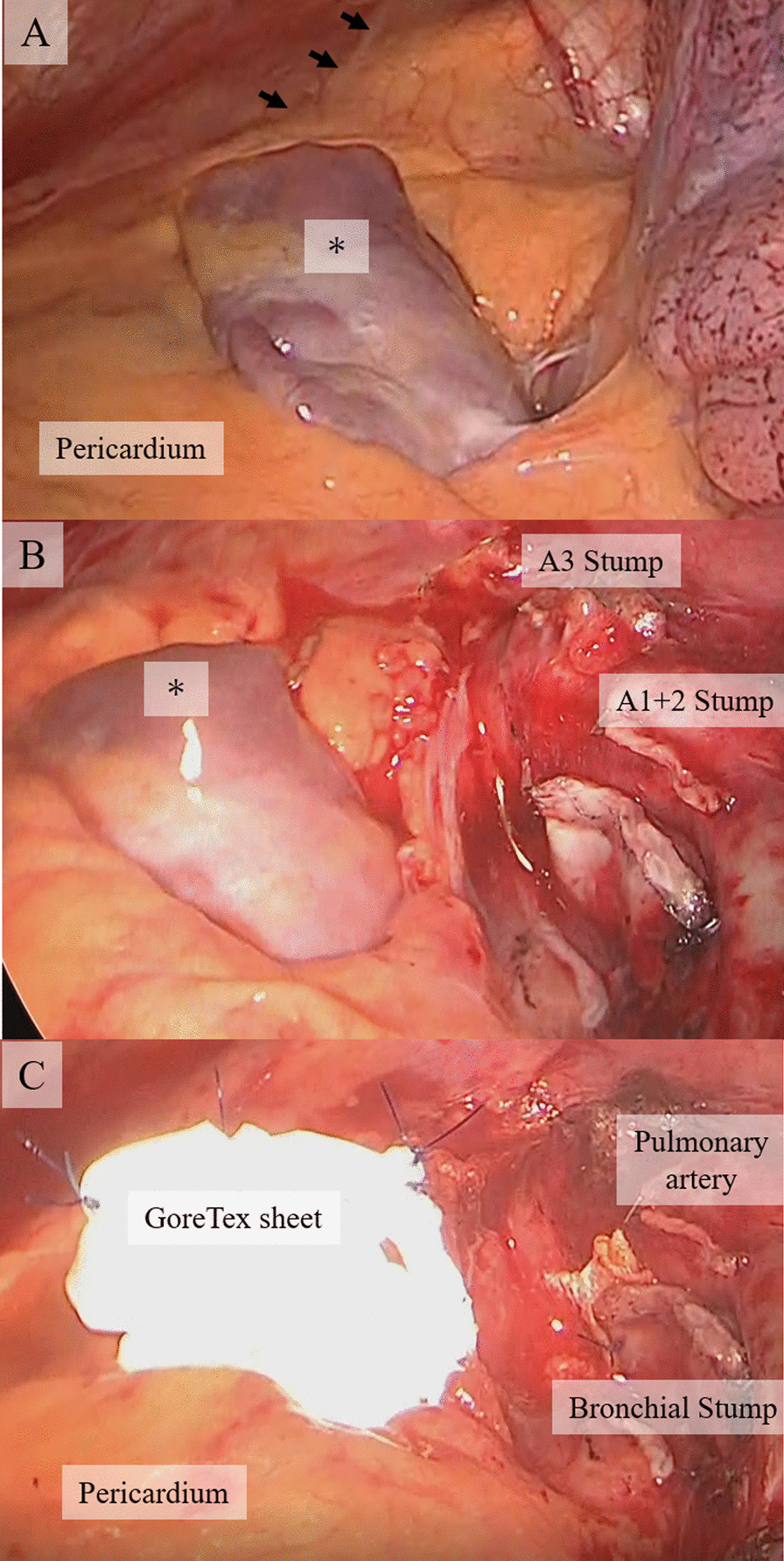
Intraoperative findings. **A** The left atrial appendage (LAA) was exposed in front of the hilar region (asterisk). The phrenic nerve was seen on the ventral side of the defect (arrow). **B** After left upper lobectomy and lymph node dissection (ND2a-1). **C** The pericardial defect was closed with a 0.1 mm expanded polytetrafluoroethylene GoreTex® surgical pericardial membrane

A 74-year-old man was referred because a 32 mm-sized ground glass shadow was pointed out in the anterior segment (S3) of the left lung on chest CT taken for the purpose of medical checkup during follow-up for hypertension and renal dysfunction. He was asymptomatic and had a history of benign prostate hyperplasia with no history of heart disease. Physical findings were normal. Blood urea nitrogen was 24 mg/dL, creatinine was 1.52 mg/dL, and estimated glomerular filtration rate was 35.7 mL/min/1.73 m^2^. Chest radiography were normal. Non-contrast chest CT showed a ground glass shadow with a maximum tumor diameter of 32 mm and a solid component with a diameter of 24 mm in the left lung S3 (Fig. 1A). No abnormal findings could be pointed out in the cardiac shadow (Fig. 1B). There was no significant hilar or mediastinal lymphadenopathy. 2-deoxy-2-(18F)-fluorodeoxyglucose positron emission tomography exhibited tracer accumulation in the nodules. No accumulation suggesting regional lymph node metastasis or distant metastasis was observed. Atrial fibrillation (Af) and complete right bundle brunch block (RBBB) were observed on the electrocardiogram (ECG). Echocardiography showed that the cardiac function was maintained but the left atrium was enlarged (51 mm). Hypokinesia was observed in the apex, anterior wall, and septum. A decrease in blood flow in the left atrial appendage was observed. The surgery was performed under four port video assisted thoracic surgery. At the time of thoracotomy, the left atrial appendage (LAA) was exposed in the hilar region. Approximately 6.0 × 5.0 cm oval pericardial defect was visualized (CPPD)  (Fig. 2A). The phrenic nerve ran on the ventral side of the defect. No pleural effusion or dissemination was observed. Partial resection of the upper lobe of the left lung was performed, and adenocarcinoma was diagnosed by rapid intraoperative diagnosis. Left upper lobectomy and lymph node dissection (ND2a-1) were subsequently performed. We were concerned about cardiac hernia (CH) due to decreased cardiac bearing capacity owing to decreased lung volume and bleeding due to the contact between the LAA and bronchial stump (BS). The BS was covered with pericardial fat pad and the pericardial defect was closed with a 0.1 mm expanded polytetrafluoroethylene (ePTFE) GoreTex® surgical pericardial membrane taking care not to involve the phrenic nerve (Fig. 2B, C). In addition, the pulmonary ligaments were dissected up to the lower edge of the inferior pulmonary vein to reduce upper lobe space. The operation time was 172 min, and the patient lost little blood. Postoperative pathological examination revealed primary lung adenocarcinoma cT1miN0M0, Stage IA1. The postoperative course was uneventful. The chest drainage tube was removed on the 1st postoperative day. Edoxaban was started for Af. He was discharged 5 days after surgery, and remains well at 6-month postoperative follow up.

## Discussion and conclusion

CPD is a relatively rare congenital anomaly. Perna’s theory that premature regression of the ducts of Cuvier (common cardinal vein) during the embryonic period delays the growth of thoracic pericardial folds and does not close the thoracic pericardial foramen is predominant [6]. The male–female ratio is 3:1 and is more common in males [1], and 30% are said to have congenital anomalies in the cardiopulmonary system [2]. Of CPD, about 9% were congenital complete pericardial defects (CCPD), 70% were left-side defects (CCPD and CPPD were similar), and 4–6% were right-side defects (mostly CPPD and rarely CCPD). Diaphragmatic pericardial defects accounted for 17%, which is overwhelmingly on the left side [7].

Some cases present with symptoms such as chest pain, palpitation, dyspnea, and absence seizures. However, it is generally asymptomatic and is often found incidentally during surgery [3]. Japanese and English literature regarding CPD found during pulmonary surgery published over 40 years from 1982 to 2021 are shown (Table 1) [3–20]. There have been 32 cases (15 cases of pneumothorax, 7 cases of lung cancer, 5 cases of bronchogenic cyst, and 5 cases of others) including this case, 28 in males and 4 in females. There were 20 cases of CCPD and 12 cases of CPPD with 30 cases on the left side, 1 case on the right side, and 1 case involving both sides. All left-sided CPPDs were found on the upper ventral side of the hilar region. In cases with pneumothorax, it was possible to diagnose a partial pericardial defect preoperatively due to the development of pericardial emphysema as a complication of pneumothorax.

**Table 1:** Summary of case reports of English and Japanese literature on lung surgery with congenital pericardial defects over the last 40 years

Case	Age	Sex	Pulmonary surgery	Side	Type	Size (cm)	Preoperative diagnosis	Congenital malformation	Phrenic nerve running	Treatment for defect	Postoperative complications	References
1	18	M	PTX	B	C	−	−	−	N.D.	−	−	[8]
2	22	M	PTX	L	C	−	−	−	N.D.	−	−	
3	35	M	PTX	L	C	−	−	−	A	−	−	
4	64	M	PTX	L	C	−	+	−	N.C.	−	−	
5	24	M	PTX	L	C	−	+	−	N.D.	−	−	
6	24	M	PTX	L	C	−	+	−	N.D.	−	−	[9]
7	72	M	PTX	L	C	−	+	−	A	−	−	[8]
8	17	M	PTX	L	P	5 × 4	+	Hypospadias	N.D.	Patch (parietal pleura)	−	
9	19	M	PTX	L	P	3 × 2	+	−	N.D.	−	−	
10	17	M	PTX	L	P	5 × 4	+	−	A	−	−	
11	23	M	PTX	L	P	5 × 4.5	+	Intestinal malformation	A	Patch (pericardial fat)	−	
12	20	M	PTX	L	P	4 × 3	+	−	A	−	−	
13	56	M	PTX	L	P	3 × 2	+	−	A	Patch (pericardial fat)	−	
14	16	M	PTX	L	P	4 × 3	+	−	A	−	−	[10]
15	22	M	PTX	L	P	4	−	−	N.D.	−	−	[11]
16	22	F	Giant bulla	L	C	−	+	−	N.D.	−	Chest pain Ischemic change in the circumflex area on electrocardiography	[12]
17	53	M	Giant bulla	L	C	−	−	−	N.D.	Repair (Marlex mesh)	−	[13]
18	35	M	Bilateral bullous emphysema (post PTX)	L	C	−	−	−	Anterior mediastinum	−	−	[14]
19	22	F	Extralobar pulmonary sequestration	L	C	−	−	ASD	N.D.	−	−	[15]
20	14	F	Bronchogenic cyst (LUL)	L	C	−	−	Funnel chest	N.D.	−	Sudden death due to excessive shift of the heart to the left	[4]
21	18	M	Bronchogenic cyst	L	C	−	+	−	N.D.	N.D.	N.D.	[16]
22	15	M	Bronchogenic cyst	L	C	−	+	ASD, MVP, hypospadias	N.D.	N.D.	−	
23	32	M	Bronchogenic cyst	L	P	4 × 3	−	−	N.D.	−	−	
24	69	F	Bronchogenic cyst	R	P	N.D.	−	−	N.D.	N.D.	Paroxysmal atrial fibrillation	[17]
25	58	M	Cystic bronchiectasis (LPn)	L	P	N.D.	−	−	N.C.	−	−	[18]
26	78	M	LC(LLL)	L	C	−	−	−	N.D.	N.D.	−	[3]
27	70	M	LC(LUL)	L	C	−	−	−	N.C.	−	− (Heart deviation to the left +)	[5]
28	61	M	LC(LPn)	L	C	−	−	N.D.	N.D.	−	− (Heart extending into the left pleural cavity +)	[6]
29	74	M	LC(LUL)	L	C	−	−	N.D.	N.D.	Repair (Prolene mesh → Marlex mesh)	− (Heart deviation to the left +)	[7]
30	49	M	LC(LUL)	L	C	−	−	N.D.	N.C.	−	−	[19]
31	61	M	LC(LUL)	L	C	−	−	−	N.D.	−	− (Heart deviation to the left +)	[20]
32	74	M	LC(LUL)	L	P	6 × 5	−	−	A	Patch (GoreTex)	−	Our case

*M* male, *F* female, *PTX* pneumothorax, *LC* lung cancer, *LUL* left upper lobectomy, *LPn* left pneumonectomy, *LLL* left lower lobectomy, *L* left, *B* bilateral, *P* partial, *C* complete, *ASD* atrial septal defect, *MVP* mitral valve prolapsus, *N.D.* not described, *A* anterior of defect, *N.C.* not confirm

In cases of CCPD, pneumothorax surgery, which is considered to have a small amount of lung resection, does not show left deviation of the heart. However, left-sided deviation of the heart was observed in cases of left upper lobectomy and giant lung cyst resection. Takizawa et al. reported a case in which the coronary circumflex branch stenosis and angina symptoms appeared on the fiber bundle of the pericardial defect due to the leftward deviation of the heart after cutting the giant pulmonary cyst [12], and Honda et al. reported a case of sudden death due to excessive cardiac deviation due to repositioning after performing left upper lobe resection for an inflammatory bronchogenic cyst [4]. These cases are thought to be caused by weakened cardiac support due to decreased lung volume, and caution should be exercised in lobectomy or surgery with a similar amount of resection.

If a partial pericardial defect is found intraoperatively, there is a high risk of sudden death due to CH or pericardial restriction, and repair is necessary [21, 22]. Gassner et al. Argue that cases of death due to partial pericardial defect are due to constriction of the apex of the heart, and that incarceration does not result in death if the defect is localized to the left atrial appendage [23]. However, there are reports that the phrenic nerve was compressed by the deviation of the left atrial appendage without incarceration, causing phrenic nerve paralysis [22]. In this case, the phrenic nerve ran in front of the defect as in previous reports [8, 10]. Therefore, due to the decrease in lung volume due to lobectomy, the incarceration and protrusion of the LAA may be exacerbated and the phrenic nerve may be compressed. What is more worrisome is that movement of the organs may cause contact between the BS and the LAA and lead to perforation and bleeding, so we closed the defect. There were five reports of treatment of these defect, two defects were repaired with artificial membrane, two were repaired with pericardial fat pad (PFP), and one with visceral pleura. Of the 11 CPPD cases reported in the past, all 9 cases with size descriptions had a maximum diameter of 5 cm or less. Three of these patients underwent defect closure. In this case, PFP was used between the pulmonary artery and the BS in order to prevent perforation and bleeding due to contact. Therefore, sufficient PFP could not be collected and the defect was closed with an ePTFE GoreTex® membrane. The ePTFE membrane is known to have no adhesions to the sternum or epicardium during reoperation of cardiac surgery [24]. There is no significant difference in the risk of postoperative mediastinitis when comparing between the group reconstructed with ePTFE membrane and the group reconstructed with autologous tissue [25]. Therefore, when sufficient autologous tissue cannot be obtained, ePTFE is considered useful for pericardial reconstruction.

**Fig. 3:**
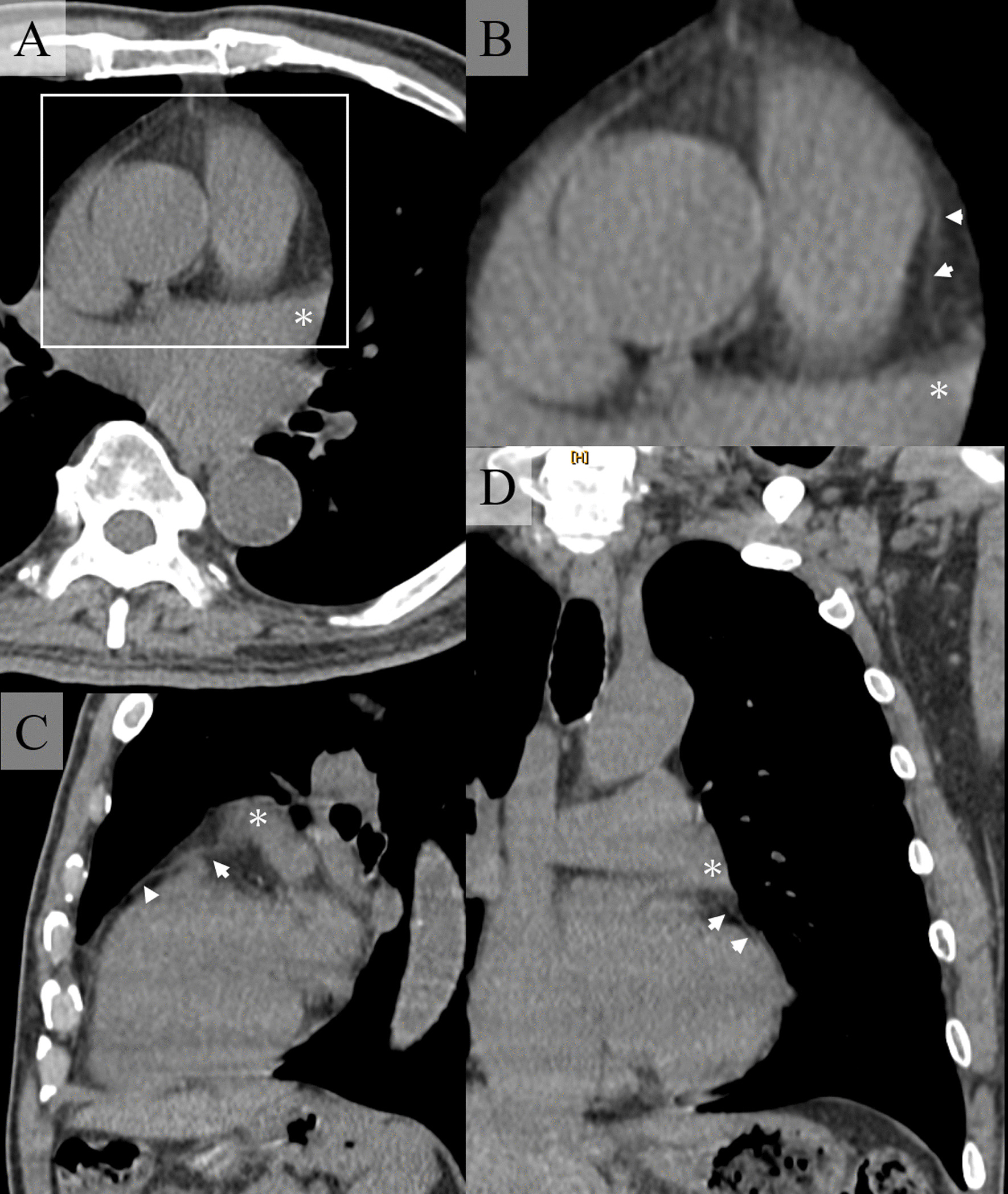
Preoperative CT reconstructed to a thickness of 1 mm. **A**, **B** Coronal section, **C** Sagittal section, **D** Horizontal section. Asterisk indicates the left atrial appendage. The white arrow head indicates the pericardium. The white square in (**A**) is the same range as in (**B**). The pericardium is seen slipping under the left atrial appendage

Recent advances in CT and MRI resolution have made it possible to make q definitive preoperative diagnoses. In a typical case, chest CT shows a finding that lung tissue intervenes between the aorta and the main trunk of the pulmonary artery due to a rotational protrusion to the left lateral side of the pulmonary artery, and a defect in the pericardium in the coronal section or horizontal section [1]. Other findings include right axis deviation and RBBB on ECG, and echocardiography shows enlargement of the right ventricular cavity and abnormal movement of the posterior wall of the left ventricle and the interventricular septum. These findings can lead to a suspicion of CPD [1]. In our case, after the surgery, we reconstructed the preoperative CT with 1 mm slices and found that the pericardium had penetrated under the LAA (Fig. 3). The thickness of the combined visceral and parietal pericardium is usually < 2 mm. Therefore, to accurately resolve the pericardium, an in-plane resolution of at least 1 mm is necessary [1]. It is not reasonable to take a 1 mm sliced CT for all lung surgery cases. However, there is no doubt that CPD should have been suspected on the basis of the ECG and echocardiography.

In conclusion, we describe a CPPD that was incidentally diagnosed during surgery for left upper lobe lung cancer. In CPPD, contact between the LAA and BS, due to movement of the lung or heart, increases the risk of bleeding after lung resection. Therefore, closure of the defect should be considered. Although it is difficult to diagnose CPPD preoperatively, CT taken with a slice thickness of 1 mm is useful for diagnosis.

## Data Availability

All data generated or analysed during this study are included in this published article.

## Abbreviations

CPD: Congenital pericardial defect
CPPD: Congenital partial pericardial defects
CT: Computed tomography
MRI: Magnetic resonance imaging
Af: Atrial fibrillation
RBBB: Right bundle brunch block
ECG: Electrocardiogram
LAA: Left atrial appendage
CH: Cardiac hernia
BS: Bronchial stump
ePTFE: Expanded polytetrafluoroethylene
CCPD: Congenital complete pericardial defects
